# Cardiac Dysfunction and Exercise Tolerance in Patients after Complex Treatment for Cranial and Craniospinal Tumors in Childhood

**DOI:** 10.3390/jcm13113045

**Published:** 2024-05-22

**Authors:** Alena Novikova, Maria Poltavskaya, Maria Pavlova, Petr Chomakhidze, Aleksandra Bykova, Nadezhda Potemkina, Maria Chashkina, Zaki Z. A. Fashafsha, Dinara Mesitskaya, Nana Gogiberidze, Anna Levshina, Ilya Giverts, Dmitry Shchekochikhin, Denis Andreev

**Affiliations:** 1 Department of Cardiology, Functional and Ultrasound Diagnostics of N.V. Sklifosovsky Institute for Clinical Medicine, I.M Sechenov First Moscow State Medical University (Sechenov University), Moscow 119435, Russia; m.poltavskaya@yandex.ru (M.P.); mgp.doc@yandex.ru (M.P.); petr7747@mail.ru (P.C.); aabykowa@yandex.ru (A.B.); na_potemkina@mail.ru (N.P.); chashkina_m_i@staff.sechenov.ru (M.C.); fashafshazaki@gmail.com (Z.Z.A.F.); aranid980@gmail.com (D.M.); nana10.11@mail.ru (N.G.); levshina.ar@gmail.com (A.L.); agishm@list.ru (D.S.); dennan@mail.ru (D.A.); 2 Federal State Institution “Scientific Research Institute for System Analysis of the Russian Academy of Sciences”, Moscow 117218, Russia; 3 World-Class Research Center «Digital Biodesign and Personalized Healthcare», I.M Sechenov First Moscow State Medical University (Sechenov University), Moscow 119435, Russia; 4 Massachusetts General Hospital, Boston, MA 02114, USA; igiverts@mgh.harvard.edu; 5 Maimonides Medical Center, New York, NY 11219, USA; 6 Pirogov’s First Moscow City Hospital, Moscow 119002, Russia

**Keywords:** brain tumors in children, risk of cardiovascular disease, radiation therapy, cardiopulmonary testing, echocardiography

## Abstract

State-of-the-art therapy improves the five-year survival rate of patients under the age of 20 with cranial and craniospinal tumors by up to 74%. The urgency of dealing effectively with late treatment-associated cardiovascular complications is rising. **Objective**: We aimed to assess echocardiographic parameters and exercise performance in subjects with a history of complex treatment for cranial and craniospinal tumors in childhood. **Methods**: the study of 48 subjects who underwent cranial and craniospinal irradiation for CNS tumors in childhood and 20 healthy age- and sex-matched volunteers was conducted. The examination included hormone studies, cardiopulmonary exercise testing, and, in the main group, echocardiography (ECHO). **Results:** In five (10.4%) patients, ECHO changes were detected after complex anti-cancer treatment: thickening and calcification of the aortic valve leaflets (2%), and reduction in the systolic LV and RV function (8% and 6%, respectively). Irradiation of various areas was a significant predictor for reduced exercise tolerance, hyperventilation at rest and upon exertion, and an increased ventilatory equivalent for carbon dioxide. Low exercise tolerance was associated with a younger age at the time of treatment initiation. Significant differences were noted between the control group and the childhood cancer survivors with endocrine disorders. **Conclusions:** The obtained data confirm the importance of regular cardiovascular and endocrine monitoring of this group of cancer survivors.

## 1. Introduction

According to the 2022 Russian Federal Service of State Statistics, about 4000–4500 cases of childhood cancer are registered annually, which is about 15–17 cases per 100,000 children [1]. There were 18.1 million estimated cancer survivors in the United States in the same year. The growing number of cancer survivors soon led to the identification of a problem: the necessity to monitor and manage acute and delayed toxicities associated with cancer treatment, as well as their effects on quality of life and premature mortality. The Childhood Cancer Survivor Study was started in 1994 to better understand these late effects, increase survival, and minimize harmful health effects [2].

Subjects with a history of malignant neoplasm in childhood and adolescence are at an increased risk of myocardial infarction, stroke, cardiomyopathy, left ventricular dysfunction, valvular disorders, and atherosclerosis, primarily because of anthracycline use and radiation therapy (RT) at the chest and neck levels [3,4,5,6]. The risk of various manifestations of cardiovascular diseases increases two- to six-fold when the effective dose of RT at the heart level exceeds 15 Gy. During craniospinal irradiation (CSR), patients may receive a spinal dose up to 36 Gy, and mean heart doses vary from 28% to 50% of spinal doses depending on RT modality [7].

Other risk factors include the features of the tumor itself, the type of surgery, the number of courses, the type of Cx, stem cell transplantation, and a combination of several treatment types. The risk of adverse effects increases with time [1]. One of the unfavorable prognostic factors is reduced exercise tolerance, which is probably due to multiple factors, including radiation and Cx toxicity [8].

Tumors of the central nervous system (CNS) hold one of the leading places among all malignant neoplasm in children and adolescents. Due to modern achievements in treatment, including surgical resections of the lesion, RT, and Cx, the five-year survival rate of patients under the age of 20 with cranial and craniospinal tumors is currently 74% [3]. Among solid tumors, the leading and second most common (20% of all malignant neoplasms in childhood) are CNS tumors in children. The incidence of CNS tumors in children from birth to 19 years of age is 3.5–4.0 per 100,000 children, with approximately 1000–1200 new cases of CNS tumors registered annually in Russia [9]. Medulloblastoma is the leading malignant CNS tumor among children, followed by malignant gliomas and anaplastic ependymomas. Less aggressive tumors include pilocytic astrocytoma and craniopharyngioma [10]. Histological analysis is used to determine the treatment strategy for CNS tumors. Modern anti-tumor treatment strategies for pediatric patients with CNS tumors involve the use of different groups of drugs with varying degrees of cardiotoxicity, leading to a high incidence of cardiovascular complications, both during active therapy and in the long term [11,12,13,14,15].

Among various complications of comprehensive treatment of CNS tumors, endocrine disorders are often identified. Growth hormone deficiency occurs in almost 100% of patients who have received more than 30 Gray of cranial radiotherapy, reproductive function disturbances are present in about half of the cases, clinically significant secondary adrenal insufficiency occurs in 3% of cases and, according to stimulation studies, in 27–46% of cases. Approximately 10–60% of childhood cancer survivors (depending on the dose of cranial radiotherapy) require replacement therapy for secondary hypothyroidism [16]. According to the study by A. Agha et al., secondary hypothyroidism was observed in 41% of cases. [17]. These findings were confirmed in the thesis “Functional status of the hypothalamic-pituitary-adrenal axis in patients who underwent comprehensive treatment for posterior fossa tumors or Hodgkin’s lymphoma in childhood and young adulthood” by Yudina A.E. [18].

The 2022 European Society of Cardiology Clinical Guidelines define many aspects of the management of cardio-oncology patients. Diagnosis of acute and subacute cardiovascular toxicity in patients receiving anticancer treatment includes ECHO with determination of ejection fraction and GLS. A separate chapter is dedicated to the long-term follow-up of patients who have survived childhood cancer. It includes risk stratification based on the total cumulative dose of anthracycline chemotherapy and the average dose of radiotherapy to the chest. Recommendations for cardiovascular monitoring include performing an ECHO every 2 years for patients at very high risk and every 5 years for those at moderate risk (IIa, B). There are no specific recommendations for patients who have undergone complex treatment for cranial and craniospinal tumors in childhood [19].

The issues of cardiovascular outcomes, risk factors, and methods of prevention in this specific cohort of patients have not been sufficiently studied. In particular, it is not known whether CSR (when the heart is exposed to a certain dose of ionizing irradiation) increases the risk of cardiac complications. The prognostic value of specific hormonal disorders has also not been sufficiently studied. Data on subclinical vascular lesions and their significance; exercise tolerance and mechanisms of its reduction; and the early predictors and possibility of the specific primary prevention of cardiovascular diseases are lacking. Thus, this study’s objective was to assess echocardiographic parameters and physical performance in subjects with a history of complex treatment for cranial and craniospinal tumors in childhood.

## 2. Materials and Methods

A single-center non-randomized non-blinded observational study was conducted. Patients were recruited from the neuro-oncology clinic of First Moscow State Medical University (Sechenov University). 

Inclusion criteria were as follows: (1) age from 16 to 40; (2) history of cranial and/or craniospinal irradiation in childhood and adolescence (up to 18 years) for central nervous system neoplasms; (3) completion of antitumor therapy ≥1 year before inclusion into the study. Criteria for inclusion in the control group were healthy volunteers who were comparable in gender and age. 

The exclusion criteria were as follows: (1) pregnancy and breastfeeding; (2) mental disorders that could interfere with conducting the study; (3) uncontrolled endocrinedisorders; (4) recurrence of malignancy; (5) new malignancy; (6) confirmed diagnosis of cardiovascular disorder before the start of antitumor treatment; (7) acute infectious and inflammatory diseases; (8) severe comorbidities.

This study was conducted following good clinical practice and the Helsinki Declaration. The protocol was approved by the Ethics Committee of Sechenov University. The study is registered at the clinicalTrials.gov website under number NCT05641636. Prior to inclusion, all subjects signed the informed consent form. Those underage signed the form in the presence of their parents. 

Information regarding age, gender, and medical history was obtained by questionnaires. History of hypertension, current smoking, diabetes, or other pre-existing medical conditions was verified by a physician during the clinical visit. 

The control group included 20 sixth year medical students of Sechenov University without any known cardiovascular risk factors and no clinical evidence of heart, lung, renal, liver, or systemic disease. 

The examination included history taking, physical examination, a 12-lead electrocardiogram, and a cardiopulmonary stress test (CS-200 Office, Schiller, Switzerland)). A hormonal profile (thyroid-stimulating hormone, follicle-stimulating hormone, growth hormone, adrenocorticotropic hormone, insulin-like growth factor 1) and transthoracic echocardiography (ECHO) were assessed on patients only in the main group.

Transthoracic echocardiography (ECHO) was performed using the “Dimension/Vivid 7 PRO” apparatus (General Electric Medical System, Oslo, Norway) with assessments of the dimensions of chambers; the thickness of the cardiac walls (end-systolic); the condition of the main vessels; the valvular apparatus; pulmonary hypertension; the ejection fraction (EF) using Simpson’s method; the time–velocity integral; and diastolic function.

A cardiopulmonary stress test was performed using by analyzing each respiratory cycle with automatic data, averaging every 30 s, until participant reached the criteria for stopping the test and/or had complaints with a severity of ≥7 points according to the 10-point Borg scale. Respiratory exchange ratio (RER, defined as VCO_2_/VO_2_ ratio) was ≥1.0. Various loading protocols were applied—BRUCE, modified BRUCE, and Naughton—based on the physical conditions of the subjects. A standard BRUCE protocol consists of 7 three-minute stages with increased speed and incline of the treadmill at each stage, with the first stage starting at 1.7 miles/hour and 10% incline. A standard modified BRUCE protocol consists of 7 three-minute stages with constant speed and increased incline of the treadmill at each stage, with the first stage starting at 1.7 miles/hour and 0.0% incline. A standard Naughton protocol consists of 7 three-minute stages with constant speed and increased incline of the treadmill at each stage on 2.5%, with the first stage starting at 1.0 miles/hour and 0.0% incline. Exercise capacity was determined based on METs. Threshold values for exercise tolerance: low < 3.9 METs, intermediate 4.0–6.9 METs, or high 7.0–10.0 METs. The following were also assessed: peak oxygen consumption (VO_2_peak); oxygen consumption after reaching the anaerobic threshold (AT); minute ventilation (MV); produced CO_2_ volume; and CO_2_ ventilatory equivalent (VE/VCO_2_). The main contraindications for stress testing were standard contraindications, including severe neurological disorders associated with surgical treatment of cancer [20,21]. As a control group, cardiopulmonary stress test data of 20 healthy volunteers were used. Detailed comparative clinical and demographic characteristics of the main and control groups of the study have been published in a previous article [22].

### Statistical Data Processing

We statistically processed the study data using software for data analysis (Prism 9.2.0), as well as the Python programming language, to construct multiple logistic regression models. The Python language was chosen because of its ability to quickly process a large amount of data and its optimal performance when performing complex statistical modeling. Data archiving and preparation for analysis were carried out using Microsoft Excel 2016 MSO (16.0.4312.1000). Quantitative indicators were presented as the mean value of the study parameter (M) with a standard deviation in a 95% confidence interval. The median values were virtually similar to the mean values; therefore, for clarity, the data were presented as mean values, but the reliability of the mean difference was calculated using the median value. Qualitative indicators were presented in the form of absolute numbers and the percentage of the total number of patients in the pooled sample or in the corresponding group. The reliability of differences in the compared qualitative values was determined using the Fisher test. For quantitative parameters, the first stage tested the normality of distribution using the Anderson–Darling test, and the second stage was a comparison using the Mann–Whitney test or Student’s *t*-test. The initial test for distribution normality was also carried out before the correlation analysis—when the distribution corresponded to normal, we used the Pearson test; when the distribution was different from normal, we used the Spearman test. Multiple regression models were prepared taking into account multiple-hypothesis testing. In statistical modeling using multiple linear regression, we assessed the following: gender, age at the time of diagnosis, age at start of treatment, age at end of treatment, time after the end of treatment, radiation therapy, chemotherapy, hormonal profile, ECHO. We assessed the main effects of the model. *p* < 0.05 was considered as statistically significant

## 3. Results

**Figure 1:**
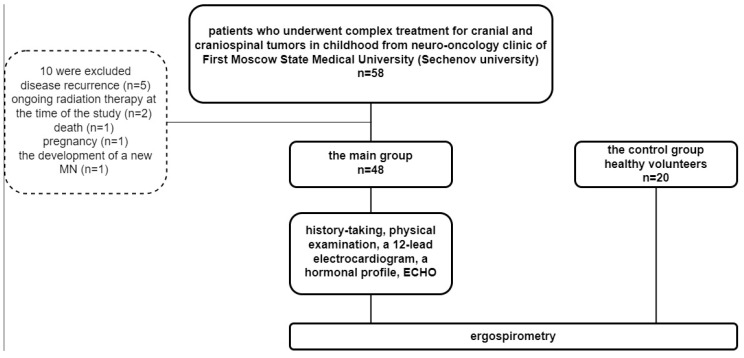
Patient inclusion.

58 childhood cranial and craniospinal survivors were screened. Ten were excluded because of disease recurrence (*n* = 5), ongoing radiation therapy at the time of the study (*n* = 2), death (*n* = 1), pregnancy (*n* = 1), and the development of a new malignant neoplasm (*n* = 1). 48 patients: 20 men and 28 women (mean age 21.7 ± 4.3 years) (Table 1). The control group included 20 healthy volunteers (Figure 1).

Notably, medulloblastoma was the most common cancer, followed by germinomas. The average time gap between the end of the treatment and inclusion to the study was 6.9 ± 5.4 years. Patients underwent complex anti-cancer treatment, which included surgical intervention (a total or subtotal tumor resection was performed on 40 patients, 83%), RT (cranial radiation (CR) was performed on 8 patients, 16%), CSR (40 patients, 83%), and chemotherapy (43 patients, 89%). The most common agents for chemotherapy were cisplatin and vincristine (Table 1). 

Clinical and laboratory data of the patients are continued in Table 2. One patient had a stroke 20 years after cancer treatment; other cardiovascular diseases were not detected.

### 3.1. Comparative Characteristics According to Endocrine Anomalies

The patients that had at least one endocrinological disorder (ED) (*n* = 39) differed significantly from the patients without such conditions (*n* = 9) in height (160.4 cm vs. 171.6 cm, *p* = 0.026), body weight (56.4 kg vs. 71.9 kg, *p* = 0.026), and mean blood pressure (74.3 mm Hg vs. 81.9 mm Hg, *p* = 0.033). A group of patients without ED did not differ in these parameters from the control group (height, 173.6 cm; body weight, 66.2 kg; mean blood pressure, 82.1 mm Hg.

### 3.2. Comparative Characteristics of ECHO Data in Patients after CSR and CR

Taking into consideration the fact that, during CSR, heart and large vessels are exposed to irradiation, we compared ECHO parameters after CR and CSR (Table 3) [23]. 

The mean age of five patients in whom ECHO changes were detected (Table 4) was 22 years (18–31). The mean time after the end of treatment was 10 years. Oncological treatment included craniospinal radiation therapy and chemotherapy.

These patients with ECHO abnormalities did not differ from the rest of the survivors in the type of tumor, radiation therapy doses, chemotherapy, remission duration, or age at the time of treatment initiation.

A correlation analysis in main group showed statistically significant correlations between IGF-1 and end-diastolic dimensions (EDD) (r = 0.52; *p* = 0.005); IGF-1 and EF (r = 0.38; *p* = 0.049); IGF-1 and EDV (r = 0.386; *p* = 0.038); IGF-1 and left atrial volume (LAV) (r = 0.41; *p* = 0.034); and IGF-1 and right atrial volume (RAV) (r = 0.42; *p* = 0.023).

### 3.3. Comparative Characteristics of the Main Clinical and Demographic Indicators in Patients after CR and CSR 

No differences were found in the nature of the tumor, doses of radiation therapy, chemotherapy, time of remission, or age at the start of treatment (Table 5).

Patients undergoing CSR therapy were more frequently subjected to surgical intervention compared to patients undergoing CR.

Patients who were receiving CSR had higher TSH levels: 2.7 ± 1.9 μIU/mL vs. 1.1 ± 1.3 μIU/mL (*p* = 0.022) (Table 6) 

### 3.4. Comparison of the Physical Performance Indicators in Patients after Oncological Treatment in Childhood and Healthy Individuals

Cardiopulmonary testing showed that physical performance parameters in patients who underwent anti-cancer treatment were significantly lower than those of healthy volunteers (Table 7). Furthermore, a reduction in ventilatory efficiency was observed. The patients had an increased risk of VO_2_ peak reduction—OR 1.613 (95% CI 1.1–2.22, *p* = 0.004).

The correlation analysis showed statistically significant correlations between peak oxygen consumption and cortisol levels (r = 0.48; *p* = 0.029), as well as between peak oxygen consumption (in percent) and cortisol concentration (r = 0.48; *p* = 0.027). Hyperventilation during exercise positively correlated with IGF-1 levels (r = 0.391; *p* = 0.036). CO_2_ ventilatory equivalent had a positive correlation with thyroxine (T4) level (r = 0.421; *p* = 0.008).

We compared patients with at least one endocrinological disorder (*n* = 39) and without such conditions (*n* = 9) with those in the control group. There were no significant differences in peak oxygen consumption (18.8 mL × min^−1^ × kg vs. 25.5 mL × min^−1^ × kg, *p* = 0.063), percentage of peak oxygen consumption (48% vs. 66%, *p* = 0.318), CO_2_ ventilatory equivalent (30.8% vs. 25.4%, *p* = 0.172), and MET loads (6.7 METs vs. 8.6 METs, *p* = 0.125), both between patients with and without ED and between patients without ED and the control group (VO_2_ peak 25.5 mL × min^−1^ × kg vs. 30.3 mL × min^−1^ × kg, % of VO_2_ peak 66% vs. 85.8%, VE/VCO_2_ 25.4% vs. 23.6%, METs load 8.6 vs. 8.9 METs (ns)). We have noted significant differences when comparing the group of patients with endocrinological disorders and the control group: peak oxygen consumption (18.8 mL × min^−1^ × kg vs. 30.3 mL × min^−1^ × kg), percentage of peak oxygen consumption (48% vs. 85.8%), CO_2_ ventilatory equivalent (30.8% vs. 23.6%), and MET load (6.7 METs vs. 8.9 METs) (*p* < 0.0001).

### 3.5. Independent Predictors of Reduced Physical Performance and Ventilation Efficiency 

In statistical modeling using multiple linear regression, we assessed the following: gender, age at cancer diagnosis, age at start of treatment, age at end of treatment, number of years since end of treatment, radiation therapy received to various areas, various chemotherapy drugs, hormonal profile, ECHO parameters (Table 8). 

Lower exercise tolerance (according to METs) was associated with a younger age at the start of treatment. Statistically significant predictors of reduced peak oxygen consumption were not identified. Hyperventilation at rest and during exercise was associated with a higher total cranial dose, and reduced ventilation efficiency had associations with a higher total cranial dose and a dose to the tumor bed.

## 4. Discussion

We performed echocardiography and cardiopulmonary exercise testing in 48 survivors of childhood CNS tumors (mainly medulloblastoma) aged 16 to 40 years (mean 21.7 ± 4.3 years), ≥1 year (mean 6.9 ± 5.4 years) after the completion of combined treatment that included surgery in 83.3%, cranial or craniospinal irradiation in 100%, and chemotherapy in 90% of patients. A total of 81% of the patients had endocrine disorders (somatotropic insufficiency, hypogonadism, hypocorticism, hypothyroidism), and received corresponding replacement therapies. In some cases, the growth hormone replacement was initiated with delay due to oncological considerations. Cardiopulmonary test data were compared to the data of 20 healthy age- and sex-matched volunteers.

No patients had arterial hypertension, obesity, or diabetes mellitus. One had a history of ischemic stroke. Compared to the control group, the patients exhibited reduced stature, likely due to endocrine insufficiency.

ECHO abnormalities were found in five (10.4%) patients: impaired LV and RV systolic function (decreased velocity time integral, GLS, and TAPSE, borderline LVEF) in four patients, and calcification of the aortic valve in one patient. All of the mean ECHO characteristics (heart chamber dimensions and volumes, LV and RV systolic function, LV diastolic function) were within the normal ranges, with no difference between the patients after cranial or craniospinal irradiation. We did not find any associations of the ECHO parameters with the dose of radiotherapy. There were modest or weak but statistically significant correlations between LVEF and heart chamber volumes and IGF-1 serum concentration, suggesting that echocardiographic changes could relate to somatotropic insufficiency and secondary hypothyroidism. 

The majority of the brain tumor survivors demonstrated decreased exercise capacity. A total of 62.5% had reduced peak oxygen consumption, 47.9% had a reduced anaerobic treshold, and 54.2% had impaired ventilatory efficiency. Mean peak oxygen consumption and exercise PetCO_2_ were significantly lower and VE/VCO_2_ was significantly higher than in the control subjects. We found weak correlations of exercise capacity with serum cortisol and ventilatory indicators with IGF-1 and T4, but they did not have significant predictive value. According to the multiple regression model, only the higher irradiation dose and the younger age at the start of treatment were independently related to exercise intolerance and impaired ventilatory efficiency.

Although the mechanism of RT-induced cardiotoxicity is not fully understood, it is hypothesized that radiation-triggered inflammatory reactions, such as the release of tumor necrosis factor, interleukins, and transforming growth factor, might cause diffuse interstitial fibrosis [7,24,25]. Late dose-dependent myocardial fibrosis is well described mostly after mediastinal irradiation [4,25]. During craniospinal radiation therapy, heart and major vessels are also exposed to some dose of radiation. A retrospective single-center study found reduced longitudinal strain (GLS) both less and more than 12 months post craniospinal radiation in patients treated for CNS malignancies from 1986 to 2018. As in our study, no association was found between the radiation dose/type and observed cardiac effects, possibly due to the small sample size and relatively low heart dose. [23]. Hummel YM et al. demonstrated decreased cardiac volumes and compromised LV systolic and diastolic function following cranial irradiation. Like in our study, these changes could be linked to diminished insulin-like growth factor levels. Growth hormones play a critical role in the structural integrity and function of the myocardium; deficiencies in growth hormones adversely impact cardiac health [26,27]. Duerr RL et al. reported that cranial irradiation for tumors of the central nervous system can lead to structural and functional changes in the heart. A possible mechanism leading to these changes is disruption of the hypothalamic–pituitary system, which can lead to growth hormone deficiency. Although a decreased left ventricular size is not considered a clinical problem, the diastolic function may be slightly reduced. However, the combination of these two factors may limit left ventricular filling, which, in turn, may reduce the cardiac output and exercise performance [28].

Exercise intolerance in cancer survivors presents a well-known problem but its mechanisms are still under discussion. Among 1041 patients, at least 10 years post-cancer treatment, K Ness et al. demonstrated reduced peak oxygen consumption in 63.8% of patients who had received cardiotoxic chemo- and/or radiotherapy and in 55.7% of those who had not. Low functional capacity was associated with increased mortality (HR = 3.9; 95% CI: 1.09 to 14.14) [29].

Growth hormone deficiency in adults leads to a reduction in muscle strength and endurance. Colao A et al. described the roles of growth hormones and IGF-1 in maintaining cardiac structure and function. Cardiovascular risk in growth hormone deficiency is associated not only with the lack of direct effects of growth hormones and IGF-1 on the heart and endothelium but also with low physical activity [30,31,32]. According to the meta-analysis of Zhang S et al., growth hormone treatment in patients with growth hormone deficiency promotes the improvement of overall vital signs and quality of life, enhancement of heart rate, reduction in diastolic blood pressure, and increase in VO_2_peak and ventilation efficiency [33].

## 5. Limitations

There are some limitations to this study, including a small sample size of patients who underwent combined treatment for cranial and craniospinal tumors in childhood, as well as the control group. The small sample size was determined by the scarcity of eligible patients. We did not perform longitudinal analysis to show changes over time. We do not have information about the cardiovascular status of the patients before treatment, since, at that time, they had no cardiovascular symptoms and signs according to the anamnesis and medical records.

## 6. Conclusions

Young patients who underwent combined treatments for central nervous system tumors with cranial or craniospinal radiotherapy in childhood exhibited a significant reduction in exercise tolerance and ventilatory efficiency related to the higher cranial and tumor radiation doses and to the younger age at the start of treatment. A total of 8.3% of them had echocardiographic evidence of left and right ventricle systolic dysfunction. Endocrinological disorders such as somatotropic insufficiency and secondary hypothyroidism were highly prevalent in the tumor survivors and, although on replacement therapy, they likely contributed to reduced exercise tolerance and cardiac function. Clarifying the mechanisms and prognostic value of these changes requires further investigation. Our findings highlight the necessity of the regular monitoring of cardiovascular and hormonal functions of this group of cancer survivors.

Patents: Patent RU №. 2023621458 from: 11 May 2023 “Database for the study of cardiac pathology and risk factors in patients who underwent complex treatment for cranial and craniospinal tumors in childhood”.

## Figures and Tables

**Table 1:** Characteristics of patients who underwent treatment for cranial and craniospinal tumors in childhood.

Parameter	Value
Age at the time of diagnosis, years	13.6 ± 4.3
Mean time after diagnosis, years	7.9 ± 5.3
Mean time after the end of treatment, years	6.9 ± 5.4
Pathological diagnosis, *n* (%):	
- Medulloblastoma	25 (52.1)
- Astrocytoma	5 (10.4)
- Germinomas	8 (16.7)
- Primitive tumors	3 (6.2)
- Other types of tumors	7 (14.5)
Hormonal abnormalities, *n* (%):	39 (81.3)
- Somatotropin insufficiency	32 (66.7)
- Hypocorticism	25 (52.1)
- Hypothyroidism	28 (58.3)
- Hypogonadism	28 (58.3)
Previous treatment, *n* (%):	
- Cx	43 (89.6)
- RT	48 (100)
- Gamma knife	1 (2.1)
- Surgery	40 (83.3)
Combination treatment, *n* (%):	
- Surgery + Cx + RT	37 (77.1)
- Cx + RT	6 (12.5)
- Surgery + RT	3 (6.3)
- RT	2 (4.2)
Antitumor agents, *n* (%):	
- Carboplatin	3 (6.4)
- Cisplatin	31 (64.6)
- Etoposide	11 (22.9)
- Ifosfamide	6 (12.6)
- Cyclophosphamide	6 (12.6)
- Temodal	9 (18.7)
- Lomustin	28 (58.3)
- Vincristine	29 (60.4)
- Cytosar	1 (2.1)
Mean number of chemotherapy cycles, *n*	9.1 ± 6.9
Mean effective dose of RT depending	
on the area of exposure, Gy:	
TCD	32.9 ± 9.9
TCSD	33.9 ± 3.6
3D conformal RT-CSA	35.2
3D conformal RT boost	60.0
3D conformal RT—metastases	40.0
TTBD	50.2 ± 9.7
PCFTD	58.1 ± 13.9
Dose for metastases	36.4 ± 18.0

Three-dimensional conformal RT-CSA: 3D conformal radiation therapy of the craniospinal area; 3D conformal RT boost: 3D conformal radiation therapy boost; 3D conformal RT—metastases: 3D conformal radiation therapy for metastases; TCD: total cranial dose; TCSD: total craniospinal dose; TTBD: total tumor bed dose; PCFTD: posterior cranial fossa, total dose.

**Table 2:** Clinical data and risk factors.

Parameter	Main Group (*n* = 48)
Men, *n* (%)	20 (42)
Height, cm	162.5 ± 12.9
Bodyweight, kg	59.3 ± 17.7
Body mass index, kg/m^2^	22.0 ± 4.2
Smokers, *n* (%)	1 (2.1)
Mean BP, mm Hg	75.8 ± 8.8
Heart rate at rest, bpm	81.3 ± 12.7
Total cholesterol, mmol/L	5.4 ± 1.3
LDL cholesterol, mmol/L	3.2 ± 1.0
HDL cholesterol, mmol/L	1.5 ± 0.5
Triglycerides, mmol/L	1.2 ± 0.6
Dyslipidemia *, *n* (%)	27 (56)

LDL cholesterol: low-density lipoprotein cholesterol, HDL cholesterol: high-density lipoprotein cholesterol; * dyslipidemia was defined as the presence of at least one lipid parameter outside the normal range.

**Table 3:** Characteristics of ECHO data in patients after CSR and CR.

Parameter	Patients after Cranial Radiation Therapy (*n* = 8)	Patients after Craniospinal Radiation Therapy (*n* = 40)	*p*-Value
LVEF, %	62.0 ± 5.7	64.4 ± 4.6	0.406
VTI, %	19.5 ± 2.9	17.9 ± 2.4	0.104
GLS, %	20.7 ± 1.0	19.1 ± 3.1	0.659
ESV, mL	19.8 ± 11.1	20.3 ± 8.7	0.954
EDV, mL	63.7 ± 24.2	60.7 ± 17.6	0.853
LA, mL	33.4 ± 12.2	33.9 ± 10.2	0.843
RA, mL	24.2 ± 7.6	23.4 ± 7.2	0.540
RV, cm	2.4 ± 0.3	2.5 ± 0.4	0.974
E	90.6 ± 13.3	90.5 ± 18.7	0.947
A	60.0 ± 17.5	59.5 ± 14.1	0.881
E/A	1.5 ± 0.4	1.5 ± 0.3	0.839
TAPSE, cm	1.9 ± 0.2	1.9 ± 0.2	0.818
E/E’med	7.5 ± 4.3	7.6 ± 5.0	0.971

LVEF: left ventricular ejection fraction, VTI: time–velocity integral, GLS: global longitudinal deformation, ESV: left ventricular end-systolic volume, EDV: left ventricular end-diastolic volume, LA: left atrium, RA: right atrium, RV: right ventricle, E/A: ratio of peak early diastolic flow over peak late diastolic flow, E/E’med: ratio of the rapid LV filling at the movement speed of the fibrous ring of the mitral valve (using tissue doppler), TAPSE: tricuspid annular plane systolic excursion.

**Table 4:** Changes in ECHO parameters after oncological treatment in childhood.

Parameters	Patient 1	Patient 2	Patient 3	Patient 4	Patient 5
Type of tumor	Germinoma	Medulloblastoma	Germinoma	Medulloblastoma	Anaplastic ependymoma
Type of treatment	Surgery + Cx + RT	Surgery + Cx + RT	Cx + RT	Surgery + Cx + RT	Surgery + Cx + RT
RT, Gy	total cranial dose—56.1 Gy, spinal dose—47.1 Gy, tumor bed dose—77.5 Gy	craniospinal dose 35 Gy, posterior cranial fossa, total dose 55 Gy	total cranial dose—24 Gy, tumor bed dose—54 Gy	total cranial dose—31 Gy, spinal dose—32 Gy, tumor bed dose—49 Gy	total cranial dose—35 Gy, spinal dose—35 Gy, posterior cranial fossa, total dose—55 Gy
Cx	Platinum drugs, alkaloids, alkylating drugs	Platinum drugs, alkylating drugs	Platinum drugs, alkaloids	Platinum drugs, alkaloids, alkylating drugs	Platinum drugs, alkylating drugs
Indicators of physical performance	Reduced VO_2_ peak 20, 9 mL × min^−1^ × kg % VO_2_ peak 52%, AT 11.2 mL × min^−1^ × kg	Reduced VO_2_ peak—13 mL × min^−1^ × kg % VO_2_ peak—28%, AT—12.2 mL × min^−1^ × kg	Reduced VO_2_ peak—11.3 mL × min^−1^ × kg % VO_2_ peak—20%, AT—8.8 mL × min^−1^ × kg	Reduced VO_2_ peak—16.7 mL × min^−1^ × kg % VO_2_ peak—37%, AT—13.4 mL × min^−1^ × kg	Reduced VO_2_ peak 25.5 mL × min^−1^ × kg % VO_2_ peak—74%, AT—15.1 mL × min^−1^ × kg
Changes in ECHO parameters	TAPSE—1.6, VTI—12%, EF—54%	TAPSE—1.6, VTI—14%, EF—66%, GLS—16%	TAPSE—1.7 VTI—14%, EF—51%	TAPSE—1.4, VTI—13.4%, EF—55%, GLS—12.5%	Calcification of the aortic valve

RT: radiotherapy, Cx: chemotherapy, VO_2_ peak: peak oxygen consumption, % VO_2_ peak: peak oxygen consumption in % of normal, AT: anaerobic threshold, GLS: global longitudinal deformation, TAPSE: tricuspid annular plane systolic excursion VTI: time–velocity integral.

**Table 5:** Comparative characteristics of the main clinical and demographic indicators in patients after CR and CSR.

Parameter	Patients after Cranial Radiation Therapy (*n* = 8)	Patients after Craniospinal Radiation Therapy (*n* = 40)	*p*-Value
Age, years	21.7 ± 4.4	21.7 ± 4.4	0.946
Age, years (min–max)	18–30	16–33	
Men, *n* (%)	4 (50)	18 (45)	0.999
Height, cm	161.1 ± 13.8	162.7 ± 12.9	0.694
Weight, kg	62.8 ± 17.8	58.6 ± 17.8	0.471
Age at the time of treatment, years	13.3 ± 2.9	12.1 ± 4.2	0.674
Time after treatment, years	6.9 ± 5.2	7.1 ± 5.3	0.797
Surgery, *n* (%)	4 (50)	36 (90)	0.018
Cx, *n* (%)	7 (87.5)	36 (90)	0.999
Cx courses, *n*	10.4 ± 12.4	8.8 ± 5.6	0.307
Total tumor bed dose, Gy Min–max, Gy	57.4 ± 2.9 54.0–60.0	51.9 ± 12.1 30.0–75.6	0.311
Total cranial dose, Gy Min–max, Gy	36.0 ± 15.8 24.0–54.0	35.5 ± 10.4 24.0–55.0	0.599
Mean BP, mm Hg	81.3 ± 8.3	74.8 ± 8.5	0.073
Heart rate at rest, bpm	74.0 ± 10.1	82.7 ± 12.7	0.067

Cx: chemotherapy, mean BP: blood pressure.

**Table 6:** Comparative characteristics of cardiopulmonary testing and hormonal profile in patients after CR and CSR.

Parameter	Patients after Cranial Radiation Therapy (*n* = 8)	Patients after Craniospinal Radiation Therapy (*n* = 40)	*p*-Value
AT, mL × min^−1^ × kg	14.8 ± 3.1	14.5 ± 3.6	0.999
VO_2_ peak, mL × min^−1^ × kg	22.5 ± 4.5	19.4 ± 6.5	0.305
TSH, µIU/mL	1.1 ± 1.3	2.7 ± 1.9	0.022
FSH, mIU/mL	3.9 ± 3.7	9.2 ± 11.9	0.089
Prolactin, µIU/mL	248.1 ± 204.4	333.2 ± 218.6	0.314
Cortisol, nmol/L	259.1 ± 177.1	400.1 ± 164.9	0.094
GH, mIU/L	1.4 ± 1.3	1.7 ± 1.8	0.617
ACTH, pmol/L	3.7 ± 1.7	5.7 ± 3.5	0.283
IGF-1, ng/mL	201.6 ± 130.2	131.2 ± 64.9	0.131

AT: anaerobic threshold, VO_2_ peak: peak oxygen consumption, TSH—thyroid-stimulating hormone, FSH—follicle-stimulating hormone, GH—growth hormone, ACTH—adrenocorticotropic hormone, IGF-1—insulin-like growth factor 1.

**Table 7:** Indicators of physical performance and ventilation efficiency in patients after oncological treatment in childhood and healthy individuals.

Parameter	Main Group (*n* = 48)	Control Group (*n* = 20)	*p*-Value
VO_2_ peak, mL × min^−1^ × kg	19.8 ± 6.4	30.3 ± 5.8	<0.0001
VO_2_ peak reduced, *n* (%)	30 (62.5)	5 (25)	<0.0001
VO_2_ peak, % of normal	50.6 ± 16.8	85.8 ± 11.4	<0.0001
Anaerobic threshold/ mL × min^−1^ × kg	14.5 ± 3.7	16.4 ± 3.9	0.315
Anaerobic threshold reduced, *n* (%)	23 (47.9)	5 (25)	0.204
METs	7.1 ± 2.6	8.9 ± 1.5	0.003
Reduced exercise tolerance, *n* (%)	23 (47.9)	5 (25)	0.003
PetCO_2_ rest, mm Hg	26.5 ± 4.8	28.8 ± 5.4	0.3001
PetCO_2_ rest less 34 mm Hg, *n* (%)	43 (89.5)	17 (85)	0.295
PetCO_2_ peak, mmHg	36.3 ± 5.2	40.6 ± 4.0	0.009
PetCO_2_ peak less 34 mm Hg, *n* (%)	18 (37.5)	2 (10)	0.010
VE/VCO_2_, %	29.9 ± 5.1	23.6 ± 3.6	0.044
VE/VCO_2_ elevated, *n* (%)	26 (54.2)	1 (5)	<0.0001

VO_2_ peak: peak oxygen consumption, % VO_2_ peak: peak oxygen consumption in % of normal (≥84% VO_2_peak*, Anaerobic threshold: ≥ 14 mL × min^−1^ × kg, >40% VO_2_ peak* MET: metabolic equivalent of task (*N* ≥ 7.0 METs), VE/VCO_2_ (*N* < 30): CO_2_ ventilatory equivalent, PetCO_2_ rest: partial pressure of CO_2_ in exhaled air at rest, PetCO_2_ peak: partial pressure of CO_2_ in exhaled air at load.

**Table 8:** Independent predictors of reduced physical performance and ventilation efficiency (multiple linear regression model).

Outcome	Possible Predictors	Value	Indicators|t|-Statistics	F-Statistics	*p*
Reduced tolerance, METs ^1^	Younger age at the start of treatment	0.145	2.165	4.686	0.035
Reduced PetCO_2_ at rest ^2^	Higher total cranial dose	0.042	3.089	9.543	0.003
Reduced PetCO_2_ at load ^2^	Higher total cranial dose	0.041	2.804	7.972	0.007
Reduced CO_2_ ventilatory equivalent ^3^	Higher total cranial dose Higher dose on the tumor bed	0.037 0.027	3.193 4.317	10.19 18.64	0.002 <0.0001

^1^—normal values ≥ 7.0 METs, ^2^—normal values < 35 mm Hg, ^3^—normal values < 30%.

## Data Availability

The datasets presented in this article are not readily available because the data is patented (Patent RU №. 2023621458).
